# Innate visual attraction before, during and after escape from adverse substrates in carpenter ants

**DOI:** 10.1242/jeb.250278

**Published:** 2025-07-08

**Authors:** Yusuke Notomi, Shigeto Dobata, Tomoki Kazawa, So Maezawa, Shigehiro Namiki, Ryohei Kanzaki, Stephan Shuichi Haupt

**Affiliations:** ^1^ Department of General Systems Studies, Graduate School of Arts and Sciences, The University of Tokyo, 3-8-1, Komaba, Meguro-ku, Tokyo 153-8902, Japan; ^2^ Research Center for Advanced Science and Technology, The University of Tokyo, 4-6-1 Komaba, Meguro-ku, Tokyo 153-8904, Japan; ^3^ Department of Applied Biological Science, Faculty of Science and Technology, Tokyo University of Science, 2641 Yamazaki, Noda-shi, Chiba 278-8510, Japan

**Keywords:** Visual orientation, Swimming, Walking, Substrate conditions, Landmark, Innate behaviour

## Abstract

Many animals exhibit an innate attraction to dark areas or objects, driving orientation behaviours such as beacon aiming. In ants, some species do not appear to display beacon aiming. Here, we show that in one such species, *Camponotus japonicus*, the behaviour is triggered when crossing liquid-covered surfaces, regardless of locomotor pattern and the presence of water in the liquid. Once initiated, beacon aiming persisted even after the ants transitioned from water to dry substrates, as evidenced by their reorientation towards a displaced beacon. Beacon aiming could be observed before the ants fully transitioned from a dry substrate to a liquid-covered surface: when the ants were isolated on a water-surrounded platform, attraction to a beacon emerged while they were contacting the water, before finally deciding to swim towards the beacon. Adverse substrate conditions in general appear to be a factor triggering beacon aiming as we also identified one condition (so far) in which even liquid immersion was not required for beacon aiming, namely upside-down walking. These results indicate that beacon aiming in *C. japonicus* is performed before, during and after escape from adverse substrates. Evidence that substrate conditions can alter seemingly hardwired responses suggests that insects may adjust even simple behaviours in response to environmental conditions in a more sensitive way than commonly assumed.

## Teaser

**Summary:** Carpenter ants orient towards a beacon depending on substrate conditions. Attraction to the beacon emerges before transfer to adverse substrates such as water and persists after leaving them.

## INTRODUCTION

Animals engaging in active locomotion are faced with the problem of choosing an appropriate direction in their current environment. At the lowest level, innate taxis-like behaviours are effective. Such innate behaviours are generally regarded as having less flexibility (Cazalé-Debat et al., 2024; Perry and Chittka, 2019). However, innate behaviours can be modulated by learning to adapt them in a context-dependent manner (Buehlmann et al., 2020; Gumbert, 2000; Riffell et al., 2008). Sensitivity of innate behaviours to intrinsic physiological factors allows modulation without learning (Gorostiza et al., 2016; Kuo et al., 2015; Nordio et al., 2022). The factors playing a role in the modulation of innate behaviours are far from being fully characterised despite the fact that a detailed understanding of neural mechanisms has been reached in some model systems (Bengochea et al., 2023; Grunwald Kadow, 2019; Kim et al., 2017).

Innate behaviours are generally conserved across a wide range of species and are stably induced through easily controlled stimuli (Bourin and Hascoët, 2003; Crawley et al., 1981). In model systems such as *Drosophila melanogaster*, innate behaviours have been used to establish standardised behavioural paradigms. Classical examples involving the visual system are Benzer's counter-current apparatus (Benzer, 1967), the T-maze or Y-maze light/darkness choice test (Hadler, 1965; Jacob et al., 1977) and Buridan's paradigm (Bülthoff et al., 1982; Wehner, 1972). Here, our intention was to use a similar innate behaviour with the addition of contextual factors to investigate their role in modulating innate behavioural responses.

The innate behaviour focused on in this study is ‘beacon aiming’ (Graham et al., 2003), consisting of a spontaneous orientation towards visually conspicuous dark areas or objects. Beacon aiming is often included in a less well-defined class of behaviours known as scototaxis, especially in ecological contexts. Such behaviours have been observed across almost all insect orders (summarised in Notomi et al., 2022), in other invertebrates (Bisazza and Gatto, 2021; Blevins and Johnsen, 2004; Chiussi, 2003; Cohen and Putts, 2013; Huang et al., 2005; Kopp and Gothe, 1995; Zanforlin, 1976) and in some vertebrates (Crawley et al., 1981; Hascoët et al., 2001; Maximino et al., 2010). Beacon aiming has also been reported in insects that are forced to swim (Beugnon, 1986; Miller, 1972; Schaller, 1969; Yanoviak and Frederick, 2014). In ants, beacon aiming has been documented in various species on water (DuBois and Jander, 1985; Endlein and Sitti, 2018; Gora et al., 2016; Schultheiss and Guénard, 2021) or land, and is typically interpreted as an innate response, independent of age, feeding state or caste (Buehlmann and Graham, 2022; Notomi et al., 2022). However, some ant species apparently do not exhibit beacon aiming (Collett, 2010; Heusser and Wehner, 2002; Notomi et al., 2022).

In the present study, we started with the hypothesis that adverse substrate conditions could be a trigger for beacon aiming. We chose a species that did not perform this innate behaviour in previous tests, the partially arboreal ant *Camponotus japonicus* (Fukushi et al., 1999), and showed that it does perform beacon aiming when substrate conditions are adverse, such as during partial immersion in water or when walking upside down. We then introduced experimental paradigms in which the ants had to transition from one substrate to another, i.e. from water to dry substrate and vice versa. This set-up allowed us to probe the dynamics of attraction to the beacon in individual ants before, during and after transition between substrates.

## MATERIALS AND METHODS

### Animals

Foragers of the Japanese carpenter ant (*Camponotus japonicus* Mayr 1866) were collected from Komaba I, II campuses of the University of Tokyo (35.661°N, 139.684°E; 35.662°N, 139.678°E, respectively) and were individually isolated in 50 ml centrifuge tubes. Prior to the experiments, they were acclimated under laboratory conditions with light exposure for at least 1 h. Where applicable, ants were also incubated likewise, for at least 1 h, between different experimental conditions. Only physically intact (no visible damage to legs and antennae) ants were selected for the experiments.

### General procedures

Experiments were conducted inside an opaque light blue plastic bucket, which had a base radius of approximately 29 cm, a top radius of 35 cm and a height of 33 cm, except where described otherwise. A vertical black paper rectangle (beacon) measuring 20 deg horizontally and 45 deg vertically from the effective centre of the floor was placed on the inner wall of the arena, based on prior studies (Gora et al., 2016; Maimon et al., 2008; Notomi et al., 2022). The beacon was placed in one of three defined positions, each separated by 120 deg, with trials evenly distributed across these positions. The arena was illuminated with white LEDs, and the laboratory temperature was maintained at 20–25°C. Each trial began with an ant being placed on the substrate at the centre of the arena by releasing it from a lidless 50 ml centrifuge tube, except for the upside-down walking experiment. In experiments involving swimming in liquids of high viscosity, the ants had difficulty adjusting their posture after contacting the liquid surface. We found that dropping them from a large height of at least ca. 50 cm was necessary to ensure they landed exactly ventral-side down to safely engage in swimming. When releasing them by flipping a rod-mounted lidless 50 ml centrifuge tube internally coated with fluoropolymer (Fluon^®^ PTFE-AGC), accurate placement in the centre of the arena could be achieved reliably. The behavioural sequence was recorded using an ILME-FX3 camera (Sony) equipped with a 24–105 mm F4 DG OS HSM lens (Sigma) at 59.94 frames s^−1^, until the ants reached the edge of the circular area used for analysis (arena edge), as defined for each experiment.

#### Experiment i: factors influencing attraction to a visual landmark (beacon)

**Fig. 1.**
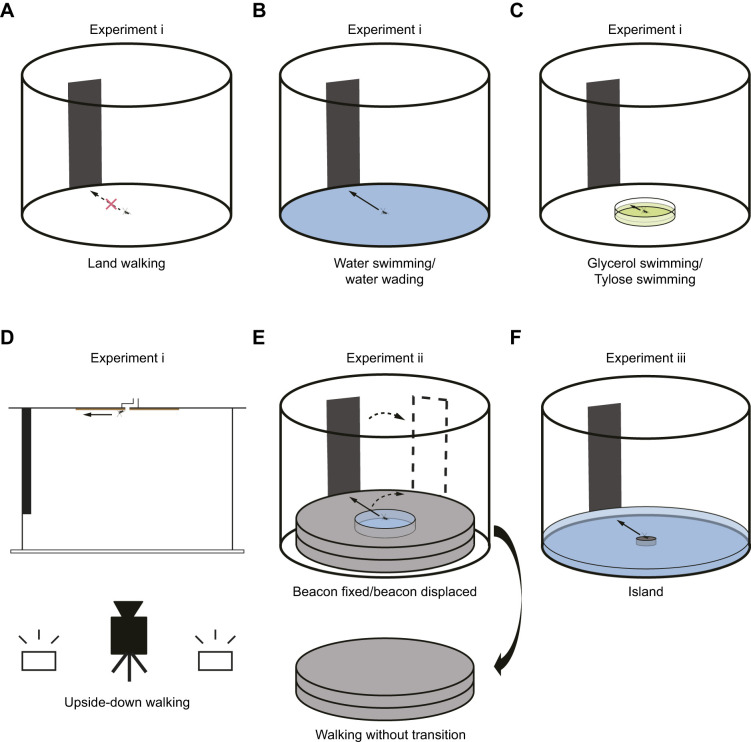
**Experimental setup.** All experiments were conducted in an arena with a black beacon (height: 45 deg, width: 20 deg, measured from the effective centre of the floor) placed against the wall. Arrows and crosses represent the overall orientation trend. (A) Basic arena used for land walking. (B) Arena filled with water for swimming (water swimming) or with shallow water for wading (water wading). (C) Arena (same as in A) with a transparent dish filled with either an 80% (v/v) glycerol and 20% (v/v) 2-propanol mixture (glycerol swimming) or a 1.0% (w/w) Tylose aqueous solution (Tylose swimming). (D) Arena with an inverted walking surface (upside-down walking) and chamber to introduce ants such that they transitioned from upside-up (on plastic) to upside-down walking (on sandpaper, brown) by themselves. The black bar indicates the beacon. The camera and illumination were positioned below the arena. (E) Arena fitted with extruded polystyrene foam inserts for transition from swimming in water to walking on a dry substrate. The insert contained a central pool filled with water (for the beacon-fixed and beacon-displaced condition). In controls, an insert of the same thickness but without the centre pool was used for identical dry walking surface conditions (for the walking-without-transition condition). The final beacon position after displacement (for the beacon-displaced condition) is shown as a dashed outline. (F) Arena (as in B) with a central platform surrounded by water (island).

To investigate whether *C. japonicus* exhibits different orientation responses on dry surfaces (i.e. land) and water surfaces, two conditions were prepared (Movie 1): ‘land walking’ – walking on a dry substrate (Fig. 1A, corresponding to Notomi et al., 2022); and ‘water swimming’ – forced swimming in the same arena filled with water (Fig. 1B, corresponding to Yanoviak and Frederick, 2014). *Camponotus japonicus* use a specific motor pattern during swimming that differs from walking locomotor patterns. As the locomotor pattern may also be a factor affecting beacon aiming, a ‘water-wading’ condition where a shallow water depth allowed the ants to walk (wade) using a walking locomotor pattern (Fig. 1B) was introduced in addition.

The water level in water swimming was set to a depth of at least 1 cm to allow smooth swimming. The water-wading condition was achieved by adding just enough water to cover the bottom surface, which allowed the ants' legs to touch the floor.

The test ants experienced all three of the conditions: land walking, water swimming and water wading. The ants were divided into two groups, with one group tested in the sequence land walking→water wading→water swimming (*n*=9 ants) and the other group tested in the sequence water wading→water swimming→land walking (*n*=10 ants). For each condition, the same 19 ants were tested with all three beacon positions, resulting in each ant being tested for 3 trials (*N*=57) per condition. Each ant was given a rest interval of at least 1 h between conditions. Ants were always only partially immersed even during swimming, but we use ‘immersion’ as a short-hand term for this condition in the following. The arena edge was defined to a radius of 20 cm.

Furthermore, the involvement of water detection in beacon aiming was assessed in a different sample of ants by replacing water with a mixture of 80% (v/v) glycerol and 20% (v/v) 2-propanol, which had a dynamic viscosity of 194.6 mPa s (kinematic viscosity: 167.1 cSt, 24°C), referred to as ‘glycerol swimming’ (Fig. 1C). As this viscosity was significantly higher than that of water (dynamic viscosity: 0.890 mPa s, 25°C; Korson et al., 1969), a control dilute aqueous solution with nearly equivalent viscosity (dynamic viscosity: 165.5 mPa s, kinematic viscosity: 166.5 cSt, 24°C) was prepared using 1.0% (w/w) Tylose (H 10,000 P2, SE Tylose, Wiesbaden, Germany) in water, referred to as the ‘Tylose-swimming’ condition.

The experimental arena consisted of an acrylic cylinder (radius, 30 cm; height, 40 cm), covered with white paper on the outside. A transparent glass Petri dish with a radius of 10 cm and a height of 2 cm, placed at the centre of the arena, was filled with liquid to a depth of approximately 1 cm. Unlike praying mantises, which require wetting by water and do not initiate swimming in liquid paraffin (Miller, 1972), ants swam reliably in the solution devoid of water.

The arena edge was defined to be at a radius of 9 cm. Each ant was tested in a single trial because of possible adverse effects, in particular of the glycerol–propanol mixture, and the beacon positions were arranged in three directions as described above (glycerol swimming: 0 deg: *n*=13 ants, 120 deg: *n=*15 ants, 240 deg: *n*=19 ants; Tylose swimming: 0 deg: *n*=15 ants, 120 deg: *n*=17 ants, 240 deg: *n*=20 ants). Ants that did not reach the arena edge within 5 min were excluded.

In addition, as an adverse substrate condition that does not employ liquids at all, ants were made to walk upside-down (‘upside-down walking’) on sandpaper. The experimental arena consisted of a white opaque acrylic cylinder (radius, 30 cm; height, 40 cm). A white acrylic board with sandpaper (#2000, DCCS-1P, Fuji Star, Okegawa City, Japan) affixed to its bottom side and with a central hole (radius, 0.4 cm) was placed atop the cylinder. The arena was illuminated from below using white LEDs, and the entire setup was positioned on a transparent acrylic board to allow video recording from below. To ensure ants could smoothly initiate upside-down walking, they were introduced via a chamber, with fluoropolymer-coated inner walls, an off-centre top hole for introduction, and a centre bottom hole connecting to the walking surface, placed above the arena (Fig. 1D).

The ants transitioned spontaneously from upside-up to upside-down walking, onto the sandpaper surface. All ants moved from the chamber to the sandpaper within 5 min. The arena edge (radius, 9 cm) was defined in alignment with the dimensions of the sandpaper (23 cm×28 cm). Each ant was tested in a single successful trial without dropping from the ceiling because of possible adverse effects of dropping, and the beacon was placed in one of three positions as described above (0 deg: *n*=16 ants, 120 deg: *n=*12 ants, 240 deg: *n*=17 ants).

#### Experiment ii: persistence of attraction to the beacon after water-to-land transition

Building on the results of experiment i, we examined whether the directional decision persisted after the transition from water to a dry substrate (equivalent to aquathlon; Fig. 1E). The experiment was conducted under three conditions: ‘beacon fixed’ – the beacon remained stationary on the arena wall; ‘beacon displaced’ – the beacon was rapidly shifted by 60 deg upon the transition of the ants from water to dry substrate; and ‘walking without transition’ – ants walked in a dry arena without a pool.

An inset composed of a light blue extruded polystyrene foam disc with a radius of 21 cm was placed on the floor of the arena (Fig. 1B). This disc was constructed by bonding two 2 cm thick boards together using waterproof adhesive. The top board featured a circular hole with a radius of 8 cm, for creating a water-filled pool. Under beacon-fixed and beacon-displaced conditions, the pool's water level was filled to the rim (i.e. depth >2 cm) to ensure a smooth transition of the ants from water to land.

Under the walking-without-transition condition, the ants were tested on a dry substrate by flipping the inset such that the side without the pool was positioned on top to act as a completely dry walking surface, due to the difference in surface structure of the polystyrene foam compared with the default arena.

The experiments were conducted in the following sequence: walking without transition→beacon fixed→beacon displaced. For each condition, the same 11 ants were tested with all three beacon positions. Each condition was separated by an interval of at least 1 h. The pool edge was defined to be at a radius of 8 cm and the arena edge at a radius of 20 cm.

#### Experiment iii: gradual emergence of attraction to the beacon before a land-to-water transition

In preliminary experiments, we observed that *C. japonicus* workers, once placed on a small, dry platform surrounded by water (referred to as an ‘island’), spontaneously left the island by swimming after a period of exploration on the platform (which would correspond to reverse aquathlon). Spontaneous exit from water-surrounded platforms has been observed previously in ants (Fielde, 1903; Wheeler, 1910; Yamaguchi, 2017, 2018). Making use of this behaviour, we investigated whether the ants orient towards a beacon prior to starting to swim and whether attraction to the beacon emerges suddenly or gradually.

A cylindrical glass cup was placed upside down at the centre of the water-filled arena to create a water-surrounded island with an effective radius of 2.75 cm (Fig. 1F). The water surface was level with the dry island surface.

Each trial began by introducing an ant into a Fluon-coated pipe with a radius of 1.5 cm, positioned at the centre of the island. The ant was released by lifting the pipe. At the end of a trial, the ant was retrieved at the defined arena edge (radius, 20 cm) before she could contact the arena wall or the beacon. The same 10 ants were tested with all three beacon positions.

The behavioural sequence was classified into four distinct phases. (1) Initial phase (prior to first water contact): this phase lasted until the mandibles crossed the defined edge of the island (radius, 2.75 cm). (2) Contact phase (before first immersion, after first water contact): starting at the end of the initial phase, this phase concluded when the mandibles crossed the defined immersion edge, represented by a circle with a radius equal to the island's radius plus two-thirds of the ant's body length. In this phase, ants contact the water mainly with their antennae and forelegs during exploration (defined as ‘water contacts’). (3) Immersion phase (before initiating swimming, after first immersion): this phase spanned from the end of the contact phase to the moment when the ant lost mechanical contact with the island. In this phase, ants protrude much more over the boundary, apparently exploring beyond for invisible gaps. This is defined as ‘water immersion’, but water contact also continues during this phase. (4) Swimming phase: beginning at the end of the immersion phase, this phase ended when the ant reached the defined arena edge (radius, 20 cm).

The moment an ant had lost mechanical contact with the island was visually confirmed to the nearest frame in the video recording. Body length was calculated as the average distance between the anterior end of the head and the posterior end of the abdomen across all frames for each ant.

### Analysis

Trajectories of the ants were obtained using DeepLabCut 2.3.8 (Mathis et al., 2018), except for upside-down walking, where UMATracker Release-15 (Yamanaka and Takeuchi, 2018) was used instead. The direction of the vector connecting the centre of the arena to the position where the ant reached the arena edge was defined as the final bearing of each trial. Similarly, the final bearings at other defined edges were also calculated. In experiments where three trials were conducted under the same condition for the same individual, all trials were pooled and treated as independent samples after the data were adjusted with reference to the corresponding beacon position, in order to eliminate potential influences of external cues and the ant's release direction that could affect outcomes depending on trial order.

Statistical analyses were conducted as previously described in Notomi et al. (2022) using Python (https://www.python.org) and the package ‘circular’ (version: 0.4–93) implemented in R (http://www.R-project.org/). Circular box-and-whisker plots were generated using a modified version of R code from Buttarazzi et al. (2018). The Rayleigh test was used to test for uniformity of circular distributions. Differences in circular distributions among experimental groups were evaluated using the Mardia–Watson–Wheeler test. All statistical tests were two-tailed and Bonferroni correction was applied for multiple comparisons.

Further data analysis details are provided in the Supplementary Materials and Methods.

## RESULTS

### Experiment i: factors influencing attraction to the beacon

Orientation towards landmarks has been studied in different species using different environmental conditions such as substrates, but a comparison of orientation under different conditions in any species appears to be lacking. To elucidate the mechanisms underlying the emergence and regulation of context-dependent behavioural differences, we investigated whether workers of *C. japonicus* orient differently on land and water surfaces.

**Fig. 2.**
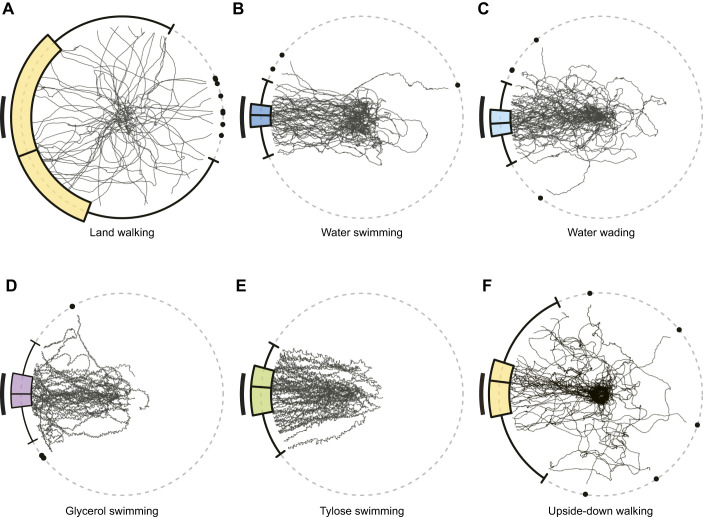
**Beacon aiming during walking and upside-down walking on a dry substrate, as well as during swimming and wading.** (A–F) Trajectories of all *N* trials and circular box-and-whisker plots of final bearings are shown for: (A) land walking (*N*=57); (B) water swimming (*N*=57); (C) water wading (*N*=57); (D) glycerol swimming (*N*=47); (E) Tylose swimming (*N*=59); and (F) upside-down walking (*N*=45). The beacon range is represented as a black arc segment on the left side of each plot. Circular box-and-whisker plots show median, first quartile, third quartile (coloured interquartile range), minimum and maximum (whiskers), and outliers (black-filled circles) of the final bearing distributions.

Under land-walking conditions, ant trajectories were relatively straight (Fig. 2; Fig. S1, Movie 1); however, their orientation appeared to be in random directions (Fig. 2A). This was reflected by a wide interquartile range spanning more than 90 deg in the circular boxplot of the final bearings, indicating no clear directional preference (Rayleigh test: 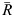
=0.19, *P*=0.140, *N*=57).

Under water-swimming conditions, except for occasional difficulties, ants adopted a distinctive swimming posture with their antennae raised above the water and a locomotor pattern characterised by extended hindlegs. During swimming, most trajectories meandered slightly but generally oriented towards the beacon or its vicinity (Fig. 2B). The interquartile range of the circular boxplot of the final bearings of water-swimming conditions overlapped with the beacon range and was relatively narrow, indicating a preference for the centre of the beacon (Rayleigh test: 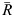
=0.95, *P*<0.001, *N*=57; mean direction: 1.3 deg, 95% confidence interval: [−1.8 deg, 4.4 deg]).

Under water-wading conditions, the distribution of final bearings (Rayleigh test: 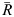
=0.97, *P*<0.001, *N*=57; mean direction: −2.1 deg, 95% confidence interval: [−5.9 deg, 1.6 deg]; Fig. 2C) was similar to that in water swimming. The final bearing distribution was not significantly different from water swimming (Mardia–Watson–Wheeler test: *W*_2_=3.12, *P*=0.629). These results suggest that the induction of beacon aiming in water is not triggered by locomotor patterns but is instead influenced by water itself in *C. japonicus*. Under land-walking conditions, neither ants that had prior experience of swimming or wading in water in the arena nor those without such experience showed significant directional orientation (Rayleigh test: land walking before water wading and land walking after water wading, 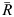
=0.17, 0.30, *P*=0.474, 0.071, *N*=27, 30, respectively; Fig. S2A,B). Likewise, significant beacon aiming in water wading was displayed by ants both with and without prior land-walking experience in the arena (Rayleigh test: water wading before land walking and water wading after land walking, 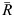
=0.95, 0.98, both *P*<0.001, *N*=30, 27; mean direction: 0.0 deg, 5.0 deg, 95% confidence interval: [−6.5 deg, 6.0 deg], [1.5 deg, 8.8 deg], respectively; Fig. S2C,D). As the ants were incubated in a dry environment for at least 1 h between conditions, these results indicate that the test conducted 1 h prior under a different condition did not significantly affect beacon aiming in the final bearings, at least.

Furthermore, the involvement of water detection in beacon aiming was assessed by replacing water with two kinds of liquid (Fig. 1C). For glycerol swimming, ants swam in a mixture of glycerol and 2-propanol. For Tylose swimming, ants swam in a Tylose aqueous solution adjusted to closely match the dynamic viscosity of the glycerol–propanol mixture. When ants were introduced into these high viscosity liquids, their smooth paddling motion was hindered. Under glycerol-swimming conditions, ants swam towards the beacon across the liquid surface, although their locomotion was impaired (glycerol swimming, Rayleigh test: 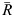
=0.95, *P<*0.001, *N*=37; mean direction: 1.5 deg, 95% confidence interval: [−4.2 deg, 6.9 deg]; Fig. 2D). Under Tylose-swimming conditions, ants also swam towards the beacon (Rayleigh test: 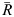
=0.96, *P<*0.001, *N*=49; mean direction: 1.0 deg, 95% confidence interval: [−3.3 deg, 5.2 deg]; Fig. 2E). No significant difference was observed in the distribution of final bearings between swimming across these two liquids (Mardia–Watson–Wheeler test: glycerol swimming versus Tylose swimming: *W*_2_=2.66, *P=*0.265). These results indicate that the presence of water is not required for the induction of beacon aiming in carpenter ants.

In addition, as an adverse substrate condition that does not employ liquids at all, ants were made to walk upside-down on sandpaper. Under this upside-down walking condition, 32/77 ants dropped from the substrate, demonstrating it is difficult to negotiate for them. This adverse substrate condition also induced beacon aiming (upside-down walking, Rayleigh test: 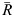
=0.72, *P<*0.001, *N*=45; mean direction: 10.0 deg, 95% confidence interval: [−2.7 deg, 24.1 deg]; Fig. 2F).

### Experiment ii: persistence of attraction to the beacon after water-to-land transition

To investigate whether the context-dependent behavioural changes are direct sensory-driven responses corresponding to environments or regulated in a more sustained manner, we next examined whether the directional decision persisted after the transition from water to a dry substrate.

**Fig. 3.**
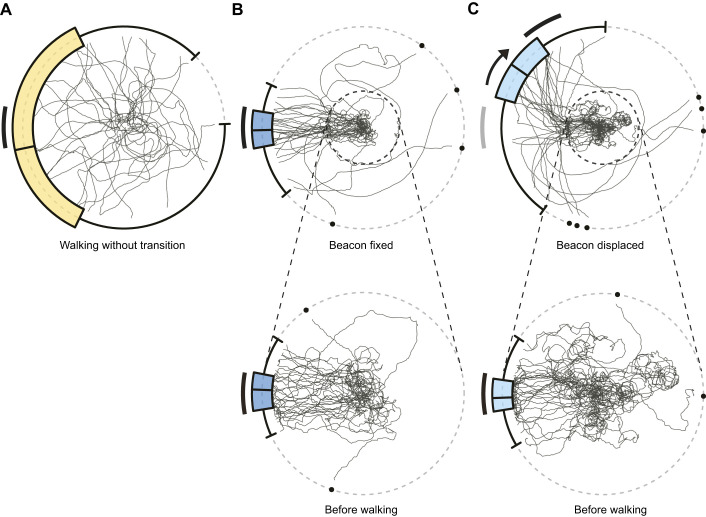
**Persistence of attraction to the beacon after water-to-land transition.** (A–C) Trajectories of all *N* trials and circular box-and-whisker plots of final bearings are shown for: (A) the walking-without-transition condition, until ants reached the arena edge (*N*=33); (B) the beacon-fixed condition, with an enlarged view of the central pool area [‘before walking’: until ants reached the central water-filled pool edge (dashed circle) swimming; *N*=33]; and (C) as for B but for the beacon-displaced condition, with an enlarged view of the central pool area (*N*=33). For the beacon-displaced condition (C), the arrow indicates the direction of the 60 deg beacon position shift. The initial beacon range is shown in grey, and the final range after displacement is shown in black.

Swimming in a pool induced beacon aiming, shown by the distributions of the final bearings on the pool edge under all conditions (beacon fixed, beacon displaced: Rayleigh test: 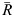
=0.85, 0.84, both *P*<0.001, *N*=33, 33; mean direction: 1.0 deg, 0.0 deg, 95% confidence interval: [−8.2 deg, 9.2 deg], [−7.9 deg, 7.3 deg], respectively; Fig. 3B,C).

Under beacon-fixed conditions, beacon aiming persisted after the transition from water to land (Rayleigh test: 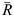
=0.78, *P*<0.001, *N*=33, mean direction: −2.7 deg, 95% confidence interval: [−10.5 deg, 5.3 deg]; Fig. 3B). No significant difference was observed between the distribution of final bearings at the pool edge and at the arena edge (Mardia–Watson–Wheeler test: *W*_2_=0.27, *P=*0.875). Under walking-without-transition conditions on the same dry polystyrene foam substrate, no significant orientation towards the beacon was observed (Rayleigh test: 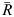
=0.28, *P=*0.145, *N=*33; Fig. 3A; Fig. S3).

Under beacon-displaced conditions, beacon aiming persisted and the directional decision was updated even after the transition from water to land, as indicated by the mean direction shifting towards the new beacon position, which was displaced by 60 deg (Rayleigh test: 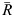
=0.61, *P*<0.01, *N*=33, mean direction: 31.5 deg, 95% confidence interval: [12.1 deg, 48.6 deg]; Fig. 3C). This shift resulted in a significant difference between the distributions of final bearings at the pool edge and the arena edge (Mardia–Watson–Wheeler test: *W*_2_=30.58, *P<*0.001) as well as between the distributions of final bearings at the arena edge under the beacon-fixed and beacon-displaced conditions (Mardia–Watson–Wheeler test: *W*_2_=30.69, *P<*0.001). The number of trials in which ants clearly did not orient towards or near the beacon after emerging from the pool was higher under beacon-displaced than under beacon-fixed conditions.

### Experiment iii: gradual emergence of attraction to the beacon before a land-to-water transition

Finally, we investigated whether the ants orient towards a beacon prior to starting to swim. The key point here is whether beacon aiming occurs before the experience of the adverse substrate condition, i.e. immersion in water, and whether internal state completely switches immediately after a single immersion.

**Fig. 4.**
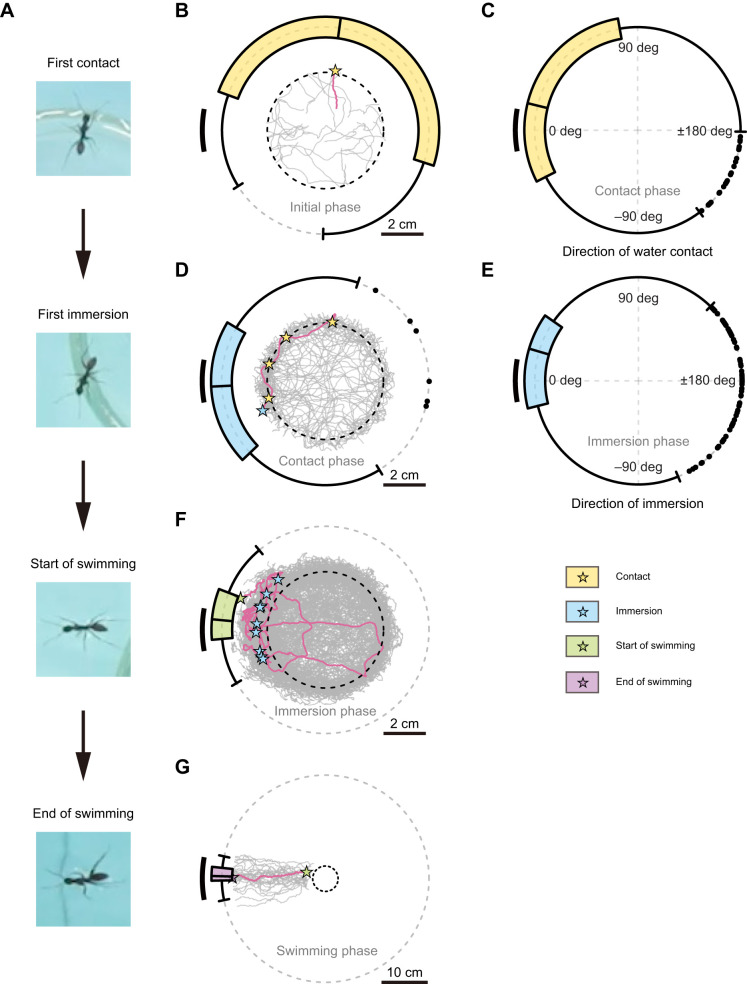
**Gradual emergence of attraction to the beacon around the land-to-water transition.** Walking on a water-surrounded platform (island, dashed centre circle) to swimming transition. (A) Representative frame images capturing key moments: first contact, first immersion, start of swimming and end of swimming. (B) Initial phase: walking trajectories on the island for this phase (until first water contact). One representative trajectory in magenta, its end point at first water contact (yellow star) displayed with a circular box-and-whisker plot of the distribution of the directions of the first water contact for all trials (*N=*33). (C) Contact phase: circular box-and-whisker plot of the distribution of directions of all water contacts (*N*=282) between first water contact and first water immersion for all trials. (D) Contact phase: conventions as in B with a circular box-and-whisker plot indicating the direction of first immersion (*N*=33). Representative trajectory in magenta, with its water contact positions (yellow stars) and its first water immersion position (blue star). (E) Immersion phase: conventions as in C, but for all water immersions (*N*=1250) until the start of swimming for all trials. (F) Immersion phase: conventions as in B, a circular box-and-whisker plot indicating the direction of swimming initiation position (*N*=33). Representative trajectory (magenta), with its immersion positions (blue stars) and its swimming initiation position (green star). (G) Swimming phase: conventions as in B but for swimming, circular box-and-whisker plot representing the direction of final swimming bearing (*N*=33). Representative trajectory (magenta), with its swimming initiation position (green star) and its final arrival position at the arena edge (purple star).

After release, the ants showed distinct phases of behaviour. During the initial phase, the ants contacted the water without a directional bias (Rayleigh test: 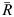
=0.05, *P*=0.917, *N*=33; Fig. 4A,B). However, a significant directional bias was evident in the distribution of all water contacts during the subsequent contact phase (Rayleigh test: 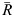
=0.27, *P*<0.001, *N*=282, mean direction: 23.2 deg, 95% confidence interval: [6.3 deg, 39.8 deg]; Fig. 4C,D; Fig. S4A). Similarly, the distribution of the first immersions was significantly non-uniform (Rayleigh test: 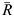
=0.34, *P*=0.033, *N*=33, mean direction: 6.0 deg, 95% confidence interval: [−33.5 deg, 53.0 deg]; Fig. 4D). However, as the 95% confidence interval of the mean direction was wider than the beacon, a possible bias for edges could not be detected. The distribution of all water immersions was narrower, with a bias towards the right edge of the beacon (Rayleigh test: 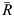
=0.66, *P*<0.001, *N*=1250, mean direction: 13.2 deg, 95% confidence interval: [10.5 deg, 15.7 deg]; Fig. 4E,F).

After several water immersions (Fig. S4B), the ants exhibited ‘pre-swimming behaviour’, a peculiar type of water immersion. This behaviour, characterised by paddling movements while maintaining contact with the shore using at least one hindleg, had intermediate characteristics between walking and swimming, complicating quantitative analysis. Most ants began swimming shortly after showing pre-swimming behaviour, with a significant orientation towards the beacon biased to its right side (Rayleigh test: 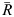
=0.94, *P*<0.001, *N*=33, mean direction: 7.0 deg, 95% confidence interval: [6.1 deg, 14.8 deg]; Fig. 4F). During the swimming phase, ants finally oriented more towards the centre of the beacon (Rayleigh test: 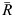
=0.99, *P*<0.001, *N=*33, mean direction: 1.4 deg, 95% confidence interval: [−1.0 deg, 3.9 deg]; Fig. 4G).

These results suggest that, while the ants showed no interest in the beacon during the initial phase, they displayed a gradually narrowing bias for the right side of the beacon during the contact phase, eventually aligning themselves with the centre of the beacon once swimming. Even after experiencing immersion once, the ants continued to explore a wide range of directions while directional choices in the beacon direction increased in frequency (Fig. S4B).

## DISCUSSION

Animals act autonomously in diverse and complex natural environments, requiring the ability to flexibly optimise their behaviour in response to dynamically changing conditions. Behavioural flexibility is not limited to the long-term changes shaped by associative learning but also encompasses other experience- and state-dependent adjustments (Gorostiza, 2018; Menzel et al., 2007; Perry and Chittka, 2019). However, such behavioural adaptations are often investigated within specific contexts and in particular species. Even for simple innate behaviours, the complete set of factors affecting them has usually not been identified.

### Beacon aiming is dependent on substrate conditions

While some ant species are known to orient towards a black beacon on land (Buehlmann and Graham, 2022; Graham et al., 2003; Macquart et al., 2006), recent findings have revealed that *C. japonicus* does not exhibit such orientation under terrestrial conditions (Notomi et al., 2022), except when trained to a landmark by appetitive conditioning (Sakiyama and Suda, 2024). In other ant species, beacon aiming on water surfaces has previously been observed (DuBois and Jander, 1985; Endlein and Sitti, 2018; Gora et al., 2016; Schultheiss and Guénard, 2021). For *Formica japonica*, there are indications for beacon aiming on both substrates (Notomi et al., 2022; Yamaguchi, 2017, 2018).

Our research shows that *C. japonicus* oriented towards a beacon on a water surface but generally did not do so on land. Beacon aiming during water surface crossing was observed not only during swimming but also while wading in shallow water. Changes in orientation depending on substrate wetness are known from a few intertidal species in specific ecological contexts (Avellanal et al., 2000; Craig, 1973; Fallaci et al., 1996). However, our ants swam towards the beacon even in a glycerol–propanol mixture that contained no water. These findings indicate that attraction to the beacon in *C. japonicus* when crossing water surfaces was triggered by immersion rather than by the use of swimming-specific locomotor patterns or detection of water itself.

The fact that *C. japonicus* exhibited beacon aiming when forced to walk upside-down on dry substrate under the risk of falling indicates that even the presence of liquid is not strictly necessary to trigger the behaviour. Dropping from arboreal environments can result in isolation from familiar surroundings and/or falling onto water surfaces, which poses a serious challenge for navigation, particularly for central place foragers (Sakiyama, 2019; Schwarz et al., 2017). Environmental factors such as wetness of substrate and temperature can affect adhesive performance (Stark et al., 2018; Thomas et al., 2023), potentially impeding locomotion. Therefore, insects adjust attachment by altering the contact areas between their tarsi and the substrate (Federle and Endlein, 2004), and change locomotor patterns dependent on substrate conditions (Dallmann et al., 2019; Duch and Pflüger, 1995; Spirito and Mushrush, 1979). By monitoring their own walking movements and detecting reductions in walking speed and/or abnormal leg rotation rates (Dürr et al., 2017; Fujiwara et al., 2017; Harris et al., 2022; Zill et al., 2004), insects are capable of detecting adverse substrate conditions. In a previous interspecific comparison of beacon aiming in ants, which employed a rather smooth substrate (Notomi et al., 2022), species-specific aversion to these substrate conditions may have contributed to beacon preference. Thus, adverse substrate conditions hampering locomotion are candidates for inducing beacon aiming in general.

In principle, like in flying insects (Franzke et al., 2022; Giraldo et al., 2018), referencing a conspicuous visual object (menotaxis) may simply help maintain a straight trajectory. At least for swimming ants, this may not be a dominant effect as ants presented with a dark vertical bar rising over a horizon well above the water surface defined by a dark/bright boundary clearly prefer aiming for the beacon rather than orienting relative to it (Gora et al., 2016). Movements across water surfaces are accompanied by risks of drowning or being preyed upon, especially for small animals (Adis, 1982; Small et al., 2013; Yanoviak et al., 2011). For ants that have fallen onto the water surface, it is most likely that a conspicuous landmark indicates the presence of land, such as a tree (Yanoviak and Frederick, 2014), whereas it may indicate the direction to the stem of a tree for ants walking upside down. Therefore, the role of beacon aiming implied by our results is that the beacon serves as a cue that assists in escaping from adverse substrate conditions.

In the context of aerial locomotion upon dropping, i.e. another adverse condition, wingless arthropods approach a vertical object as an indicator of a nearest tree (Yanoviak and Dudley, 2006; Yanoviak et al., 2005, 2008, 2009, 2011; Zeng et al., 2015, 2024). Stripe fixation (Götz, 1975; Reichardt and Wenking, 1969), a behavioural response equivalent to beacon aiming, is enhanced by wing manipulations in walking flies (Gorostiza et al., 2016). Tethered walking flies with glued wings do perform reliable stripe fixation (Bahl et al., 2013). However, without wing manipulations, substantial airflow from the ventral side appears to be required to obtain stable stripe fixation (Green et al., 2019). Thus, adverse conditions in general, including physical impairments, appear to induce or enhance stripe fixation/beacon aiming. This implies that beacon aiming in *C. japonicus* should be studied in relation to adverse factors other than mechanical substrate conditions.

Beacon aiming is also observed in contexts involving approaches to intermediate points along learned foraging routes, reducing odometric errors (Chittka et al., 1995; Graham et al., 2003), as well as in approaches to a landmark indicating a nest or known feeder location (Collett and Rees, 1997; Freire et al., 2023; Narendra et al., 2007). In the context of aerial locomotion, it may serve as an indicator of vegetative perches (Maimon et al., 2008; Munk et al., 2015; Yanoviak and Dudley, 2006; Zeng et al., 2024). Therefore, various contexts can induce beacon aiming, which itself is not always indicative of adversity. However, it remains to be explained whether these context-dependent responses are similar to reflexes or fixed action patterns triggered directly by defined stimuli or whether they are instead regulated dynamically and can be switched on and off through adjustment, for example, of factors such as adversity (Gorostiza, 2018; Herberholz and Marquart, 2012; Ronacher, 2019; Sane et al., 2020).

### Dynamics of beacon aiming related to substrate transitions

The substrate transition experiment involving transition from land to water allows the ants to choose whether they want to expose themselves to adverse substrate conditions. When observing the behaviour of our ants on the island, we found their initial orientation lacked clear directional preference, while their final exploratory decision immediately before swimming aimed for the beacon. Under normal substrate conditions, *C. japonicus* walking on land typically show no motivation for beacon aiming. However, unless an ant leaves the island, she cannot return to her nest or familiar routes, and leaving the island requires crossing the water, unless a bridge is present. Upon swimming or wading in water, the ants immediately decide to aim for a beacon. Thus, the final beacon aiming observed on the island suggests that the directional decision, normally made upon swimming or wading, was already made prior to leaving the island.

The contact phase in the exploration of the island can be interpreted as a search for a land bridge. The single visible landmark may indicate the presence of the nearest such bridge. In this phase, ants contacted water with only one or two forelegs, and a preference for the beacon direction emerged gradually. The transition to the immersion phase, in which more legs and body parts become immersed, can be interpreted as an attempt to cross a water-filled gap towards an invisible target protruding from the water. This is similar to gap-crossing behaviours involving tactile or visual sensing previously reported in other insects (Blaesing and Cruse, 2004; Pick and Strauss, 2005). In the present experiments, vision could not have been decisive, and we can suppose that gap-crossing attempts are tactile sensing for the other side of a gap. In the immersion phase, the preference for the beacon direction continued to increase during repeated gap-crossing attempts. Rather than a binary decision between beacon aiming and no beacon aiming, there was a continued, gradual increase of the preference for the beacon direction over these two behaviourally distinguishable phases. Before leaving the island, the ants exhibited ‘pre-swimming behaviour’. As they could easily return to the island as long as their hindlegs remained in contact with it, this behaviour can be regarded as intensifying attempts for gap crossing. When ants started swimming, it could not be determined whether they did so by spontaneously lifting their hindlegs or whether hindleg contact was lost once sufficient propulsive force was generated during pre-swimming behaviour. The initial swimming directions were roughly oriented towards the beacon, and they were more variable than the directions in water–land transitions. It is therefore possible that ants simply gave up on gap-crossing attempts at some point, which did not necessarily coincide with the best directional choice for beacon aiming.

In the experiment studying the water-to-land transition, the dynamics of the beacon-aiming decision are also not directly correlated with adverse substrate conditions, as ants continued to perform beacon aiming, updating their directional decision to a new, displaced beacon position even after having reached the shore. There were also trials in which ants apparently ceased beacon aiming once on dry substrate, more so when the beacon was displaced. To some extent, the movement of the beacon may have played a role, disqualifying it as a useful landmark. Such cases show that there must also be a means to switch off beacon attraction fairly rapidly. How long beacon aiming lasts after a water-to-land transition is unclear, but beacon aiming on land was abolished by 1 h incubation in a dry environment after testing beacon aiming in water (Fig. S2). Whether another persistence effect, after-fixation, as observed by Strauss and Pichler (1998), occurs under these conditions or in beacon aiming in ants in general is uncertain.

### Neural mechanisms

In insects, the central complex (CX) is a key group of neuropils involved in spatial orientation that links sensory information such as visual compass cues, wind direction and idiothetic information representing heading direction with an internal goal direction to generate steering information relayed to premotor centres (reviewed by Basu and Nagel, 2024; Collett et al., 2025; Fisher, 2022; Honkanen et al., 2019; Pfeiffer and Homberg, 2014; Varga et al., 2017). The neural architecture of the CX appears to be quite conserved across insect species (Heinze, 2024; Pfeiffer and Homberg, 2014) and both learned and innate beacon aiming of the wood ant *Formica rufa* can be explained using a realistic CX model (Goulard et al., 2021).

Information that links behaviour and neural substrates, even if only by correlation, is often restricted to *Drosophila.* In this species, flies with mutations affecting CX structure can still perform stripe fixation (Strauss and Heisenberg, 1993), and stripe fixation is immune to manipulations of the ellipsoid body (EB) heading direction system that abolish menotaxis with respect to a bright stripe (Giraldo et al., 2018; Green et al., 2019; Sullivan et al., 2019). This is similar to findings in the wood ant in which learned, mushroom-body-dependent orientation with respect to a beacon (Buehlmann et al., 2020), i.e. a learned menotaxis, reverted to innate beacon aiming following stabbing lesions in the CX (Buehlmann et al., 2023). However, optomotor responses in *Drosophila* (Akiba et al., 2020) and in the cockroach *Blaberus discoidalis* (Kathman et al., 2014) were affected by CX manipulation. Thus, an involvement of the CX in innate beacon aiming/stripe fixation cannot be excluded.

In *Drosophila*, T4 and T5 small-field motion detectors providing input to the lobula are involved in optomotor responses in stripe fixation, but at low speeds that should be relevant for walking, fixation is still possible without them (Bahl et al., 2013; Fenk et al., 2014). The circuitry in the optic ganglia allowing stripe fixation without T4/T5 neurons appears to be unidentified so far. One element that appears to be important for stripe fixation are the LC14 contralaterally projecting lobula columnar neurons (Linneweber et al., 2020), which have receptive fields with a large vertical extent and approximately 50 deg in the horizontal (Fenk et al., 2022). There are pathways from the optic ganglia to descending neurons (DNs) bypassing the CX, as at least 10% of *Drosophila* DNs innervate the optic glomeruli (Namiki et al., 2018). Some DNs (DNg02) involved in steering in response to a moving stripe in flying *Drosophila* are known (Namiki et al., 2022), but their input circuitry has not been elucidated so far.

Attractiveness of visual stimuli and valence changes in *Drosophila* could result from pairing attractive odours with the activation of octopaminergic and tyraminergic neurons, presumably affecting lobula interneurons (Cheng et al., 2019) or by activation of *Drosophila* neuropeptide F (dNPF)-containing neurons (Grabowska et al., 2018). Gorostiza et al. (2016) showed a reversal of phototaxis depending on flight ability in *Drosophila* that could be mimicked by antagonistic regulation through dopamine and octopamine. As stripe fixation was also affected by flight ability, interfering pharmacologically with dopaminergic and octopaminergic systems in *C. japonicus* could establish a link to these findings.

## Supplementary Material

10.1242/jexbio.250278_sup1 Supplementary information
