# Supplementary material for: Innate visual attraction before, during and after escape from adverse substrates in carpenter ants
Source: J Exp Biol. 2025 Jul 8;228(13):jeb250278. doi: 10.1242/jeb.250278 (PMC12276813; doi:10.1242/jeb.250278)
Supplement: Supplementary information [file jexbio-228-250278-s1.pdf]

Supplementary Materials and Methods.

The video acquired at 59.94 fps was resampled and converted to 60.00 fps before analysis. Trajectories of the ants were obtained by tracking the anterior position of their heads using DeepLabCut, except for Upside-down-walking, where the quality of the obtained videos made it impossible to reliably identify detailed body parts, and centroids were tracked instead of heads using UMATracker. When the likelihood of the estimated position calculated in DeepLabCut fell below 0.90 for any body part in a frame, the data from DeepLabCut were considered missing values and estimated by linear interpolation from adjacent frames with sufficiently high likelihood. However, the likelihood threshold was set to 0.99 for the experiment (iii), as a large manually annotated dataset consisting of 7,595 images was prepared. The accuracy of tracking was validated visually by overlaying the computed trajectory data onto the original videos and by creating labelled images for interpolated frames. To improve tracking accuracy, a total of four key points (anterior head, posterior head, anterior abdomen, and posterior abdomen) were annotated for the data in DeepLabCut. The behaviour of each ant was analysed until its mandibles (anterior position of their heads) reached the edge of the analysis area defined for each experiment or phase of an experiment. The frames where the ant entered a blind spot of the introduction chamber immediately after release were excluded from the analysis.

The vector connecting the centre of the arena to the position where the ant reached the defined edge was normalised to a unit circle, and its direction was defined as the final bearing of each trial or phase of a trial. Defined edges other than the arena edge were measured: the final bearing of swimming in experiment (ii) was defined at the first head contact with the pool edge; the final bearings of the Initial Phase, Contact Phase, Immersion Phase, and Swimming Phase in experiment (iii) were defined at the final head position in each respective phase. The orientation within the arena was defined in clockwise direction as viewed

from above. The orientations of the ants were calculated as angles relative to the centre of the beacon, set at 0°.

The resultant vector $\vec{R}$ was calculated by summing the unit vectors $\vec{u_i}$ (cos θ_i, sin θ_i) from the three trials for each ant within a condition, where θ_i represents the final bearing in trial i:

$$\vec{R} = \sum_{i=1}^{n} \vec{u_i}$$

The mean resultant length (MRL) was obtained by normalising the magnitude of the resultant vector $|\vec{R}|$ by the number of trials n:

$$MRL = \frac{|\vec{R}|}{n}$$

The mean direction $\bar{\theta}$ was calculated as the angle of the resultant vector:

$$\bar{\theta} = arctan \frac{\sum_{i=1}^{n} sin\theta_i}{\sum_{i=1}^{n} cos\theta_i}$$

The MRL is a quantitative parameter related to the degree of circular consistency in the (individual) mean direction and serves as a measure of individual variability in orientation for this study. An MRL value of 1 corresponds to no variability (i.e., a straight trajectory to the beacon), while an MRL value of 0 corresponds to maximal variability (i.e., a uniform distribution). Path straightness was defined as the ratio of the length of the final vector to the total trajectory distance for each individual trial. For comparisons made among non-circular data groups, the Mann–Whitney U test was used.

For experiment (iii), the number of water contact events was counted as the number of times the anterior head crossed the island edge. Similarly, the number of immersion events was defined as the number of times the anterior head crossed the immersion edge.

Journal of Experimental Biology • Supplementary information

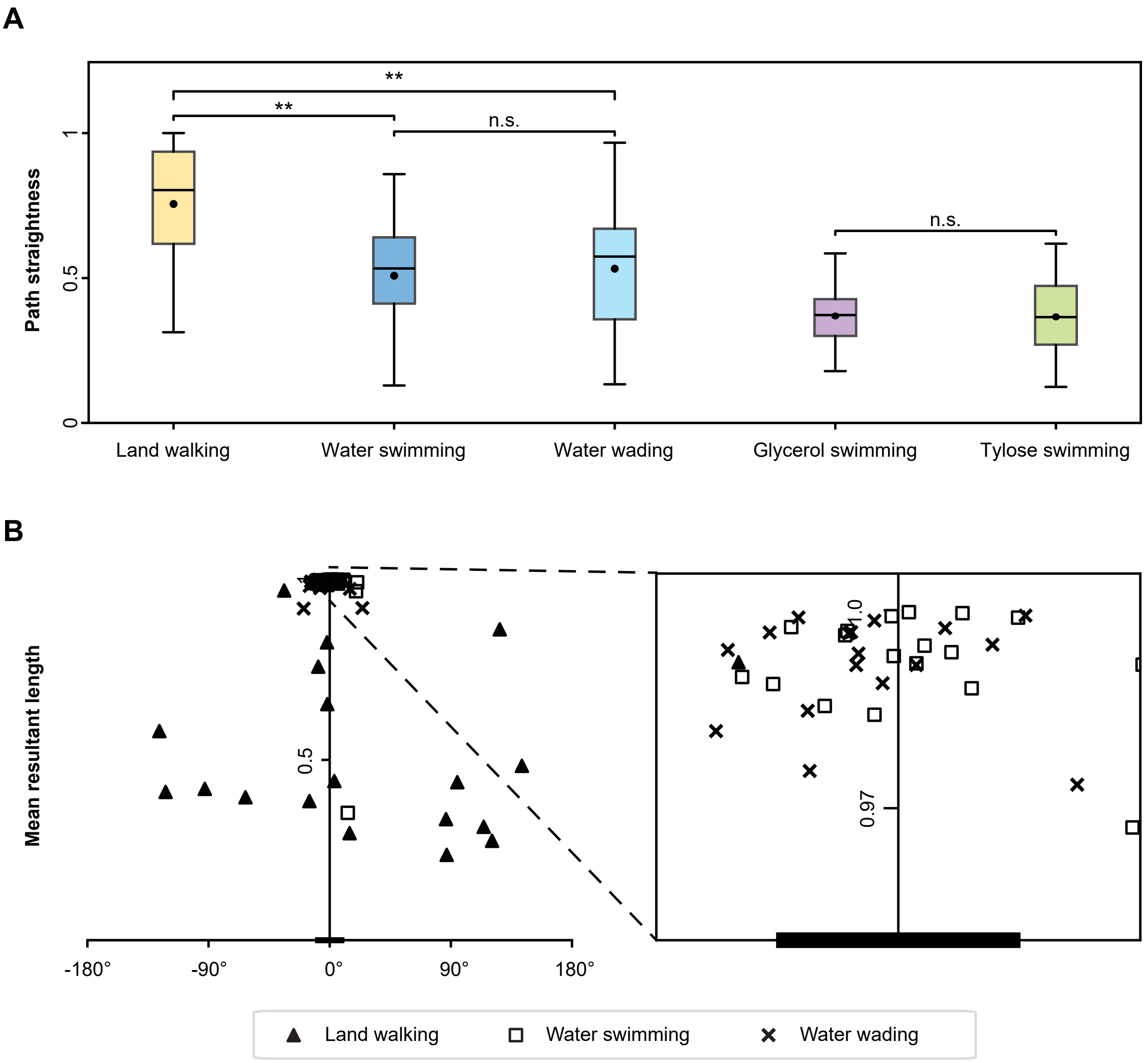

Fig. S1. (A) Path straightness across the five conditions: 'land walking'; 'water swimming'; 'water wading'; 'glycerol swimming'; 'Tylose swimming'. ** indicates a significant difference ($P < 0.01$), and n.s. indicates no significant difference ($P > 0.05$), as determined using the Mann–Whitney *U* test. Box-and-whisker plots illustrate the median, first quartile, third quartile, minimum, maximum, and mean (black-filled circles). Under land-walking conditions, the path straightness was significantly higher than that under the other two conditions (Mann–Whitney *U* test: land walking and water swimming: $U = 702$, $P < 0.001$, $N = 57$, 57, respectively; land walking and water wading; $U = 613$, $P < 0.001$, $N = 57$, 57, respectively), while no significant difference was observed between water-swimming and water-wading conditions (Mann–Whitney *U* test: water swimming and water wading: $U = 1{,}509$, $P = 0.516$). Additionally, no difference in path straightness was observed between glycerol swimming and Tylose swimming (Mann–Whitney *U* test: glycerol swimming and Tylose swimming: $U = 891$, $P = 0.897$, $N = 37$, 49, respectively). (B) Scatter plot illustrating the mean resultant length of final

Journal of Experimental Biology • Supplementary information

bearings for ants across three conditions: land walking (filled triangles, N = 19), water swimming (open squares, N = 19), and water wading (cross marks, N = 19). The MRL quantifies the extent of directional variation across trials for each ant, with values approaching 1 indicating minimal variation and strong directional consistency. When ants consistently aim for the centre of the beacon, the mean direction converges towards 0°. Scatter plots of individual trends reveal that the water-swimming and water-wading conditions exhibited minimal variation and a strong concentration around the beacon compared to the land-walking condition. Mostly low MRLs in land walking also imply that ants do not perform menotaxis with respect to the beacon at an angle specific to each individual. A black bar indicating the range of the beacon is drawn near the origin in the left figure and under the bottom of the zoomed window in the right figure.

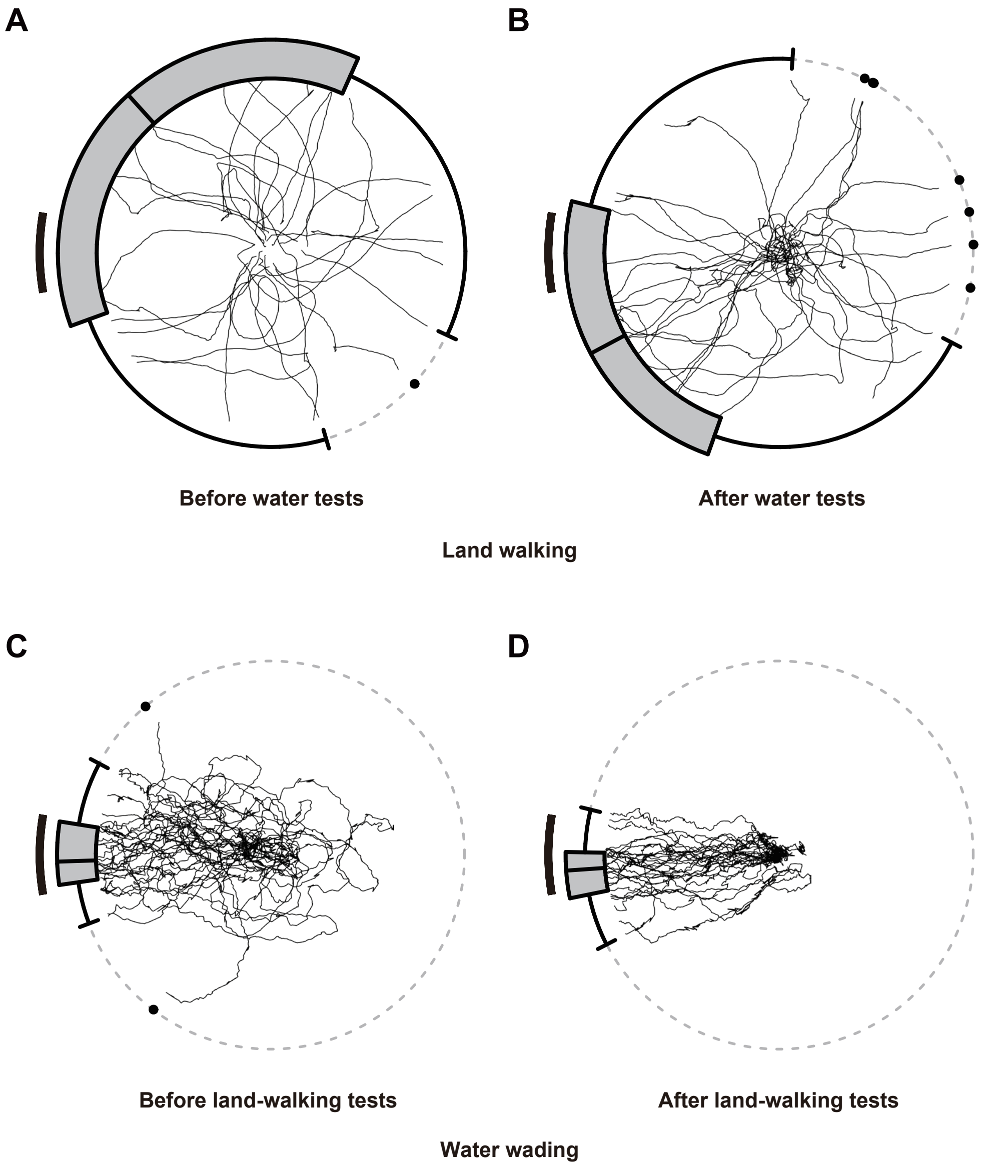

Fig. S2. (A-D) Trajectories and circular box-and-whisker plots of final bearings are shown under the following conditions: (A) land-walking condition before water-wading and water-swimming condition test ("Before water tests", $N = 27$); (B) land-walking condition after water-wading and water-swimming condition test ("After water tests", $N = 30$); (C) water-wading condition before land-walking and water-swimming condition test ("Before land-walking tests", $N = 30$); (D) water-wading condition after land-walking and water-swimming condition test ("After land-walking tests", $N = 27$).

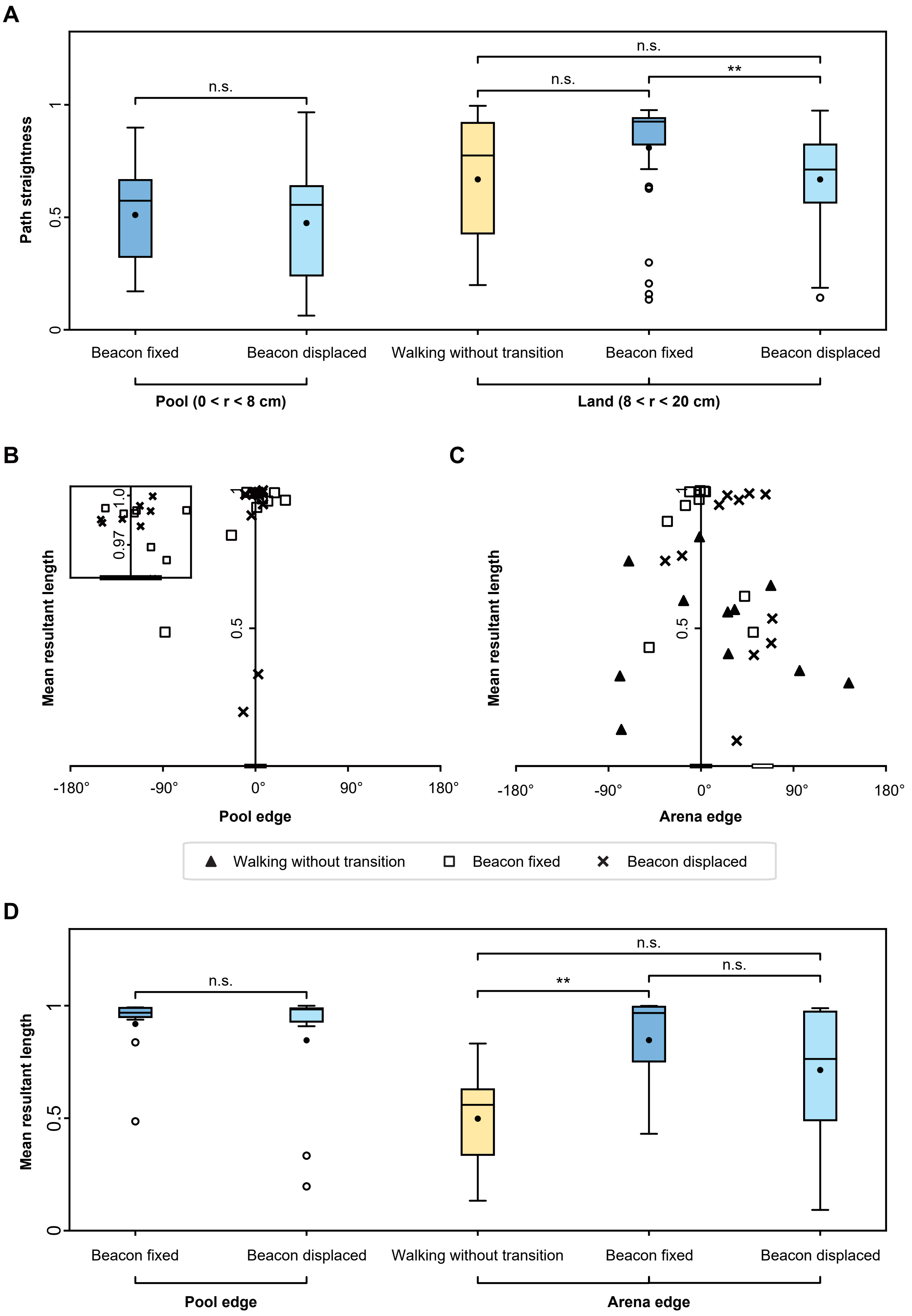

Journal of Experimental Biology • Supplementary information

Fig. S3. (A) Path straightness during swimming (Pool) and walking (Land) was analysed across three conditions: walking without transition (Land only, N = 33), beacon fixed (N = 33), and beacon displaced (N = 33). ** indicates a significant difference (P < 0.01), and n.s. indicates no significant difference (P > 0.05), as determined using the Mann–Whitney U test. During swimming, there was no significant difference in the path straightness between the beacon-fixed and beacon-displaced conditions (Mann–Whitney U test: U = 490, P = 0.491). During walking, there was no significant difference in the path straightness between walking-without-transition and beacon-fixed conditions (Mann–Whitney U test: U = 438, P = 0.175), and the beacon-fixed and beacon-displaced conditions (Mann–Whitney U test: U = 410, P = 0.086), whereas a significant difference was observed between beacon-fixed and beacon-displaced condition (Mann–Whitney U test: U = 329, P = 0.005). Box-and-whisker plots depict the median, first quartile, third quartile, minimum, maximum, mean (black-filled circles), and outliers. (B-C) Scatter plots display the mean resultant length of final bearings at the pool edge (B) and arena edge (C) for ants across the three conditions: walking without transition (filled triangles), beacon fixed (open squares), and beacon displaced (cross marks). The black bar near the origin represents the range of the beacon, and the white bar at 60° represents the range of the displaced beacon. (D) The mean resultant lengths at the pool edge and arena edge for all ants across the three conditions (walking without water, beacon fixed, and beacon displaced) is summarised. Box-and-whisker plots are presented as described in (A). At the pool edge, the swimming final bearings across all ants are not significantly different between beacon-fixed and beacon-displaced conditions (Mann–Whitney U test: U = 59, P = 0.332). At the arena edge, the walking final bearings across all ants are significantly different under walking-without-transition and beacon-fixed conditions (Mann–Whitney U test: U = 25, P = 0.002) but not significantly different between walking-without-transition and beacon-displaced conditions (Mann–Whitney U test: U = 32, P = 0.065) and beacon-fixed and beacon-displaced conditions (Mann–Whitney U test: U = 410, P = 0.086).

Journal of Experimental Biology • Supplementary information

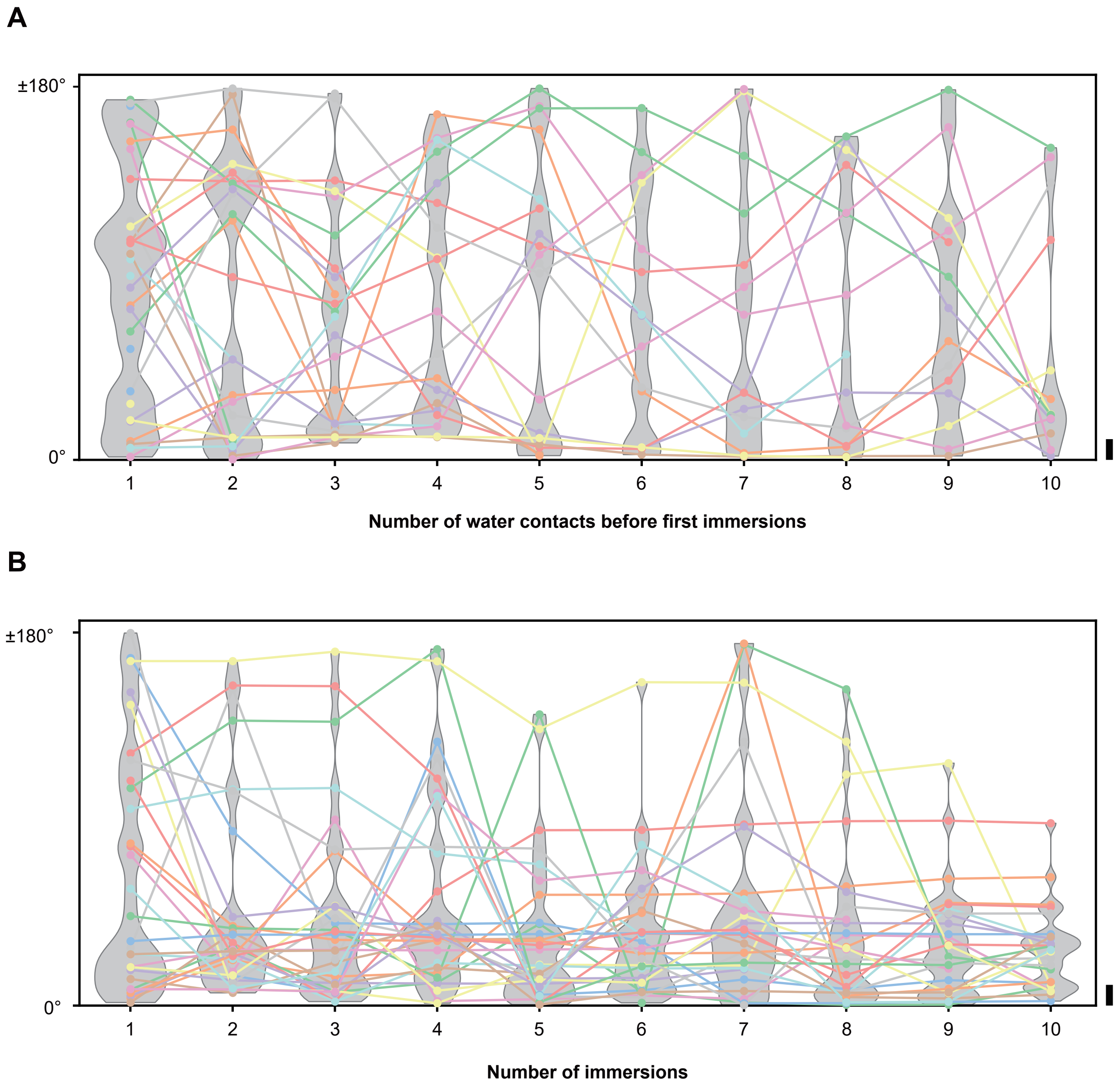

Fig. S4. (A) Distribution of directions for each water contact before the first immersion (only first 10 contacts shown). The direction of contacts in each trial is represented by filled circles, with consecutive contacts within the same trial connected by lines and shown in the same colour. The violin plots in grey are placed in the background to illustrate the distribution trends. The beacon range is represented as a black vertical on the right side of the plot. (B) Directions at immersion were plotted in the same manner as in (A), only first 10 contacts shown.

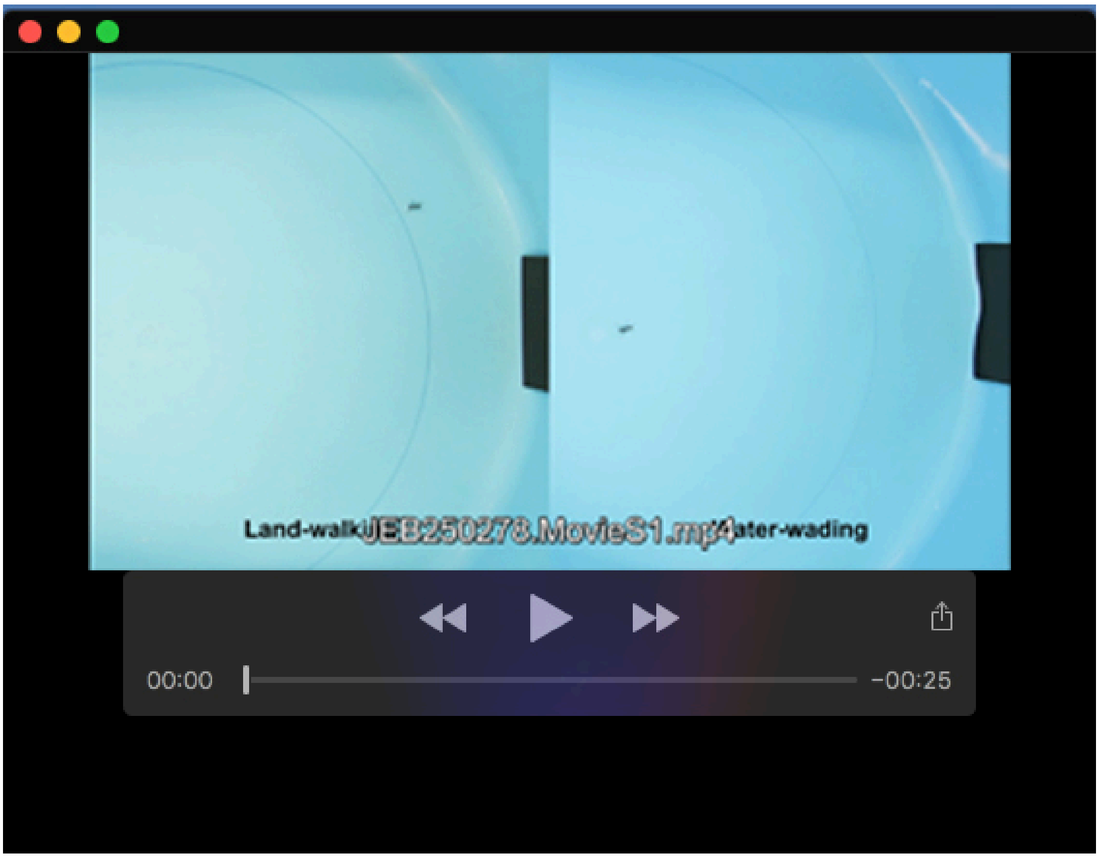

Movie 1. Examples of walking, swimming, and wading in *Camponotus japonicus*.

Journal of Experimental Biology • Supplementary information
